# Association of childhood physical and sexual abuse with arthritis in adulthood: Findings from a population-based study

**DOI:** 10.1016/j.pmedr.2021.101463

**Published:** 2021-06-24

**Authors:** Philip Baiden, Lisa S. Panisch, Henry K. Onyeaka, Catherine A. LaBrenz, Yeonwoo Kim

**Affiliations:** a The University of Texas at Arlington, School of Social Work, 211 S. Cooper St., Box 19129, Arlington, TX 76019, United States; b University of Rochester Medical Center, Department of Psychiatry, Center for the Study and Prevention of Suicide, 300 Crittenden Blvd., Rochester, NY 14642, United States; c Harvard Medical School, Department of Psychiatry, Massachusetts General Hospital/McLean Hospital, Boston, MA 02115, United States; d The University of Texas at Arlington, College of Nursing and Health Innovation, Department of Kinesiology, 500 W. Nedderman Dr., Arlington, TX 76019, United States

**Keywords:** Arthritis, Childhood physical abuse, Childhood sexual abuse, Chronic illness

## Abstract

The objective of this cross-sectional study was to investigate childhood physical and sexual abuse as factors associated with arthritis among adults from selected states in the United States. Data for this study came from the 2019 Behavioral Risk Factor Surveillance System survey. An analytic sample of 75,717 adults 18–75 years old (49.7% female) was analyzed using log-binomial regression. The outcome variable investigated in this study was arthritis, and the main explanatory variables were childhood physical and sexual abuse. Of the 75,717 respondents examined, 20,938 (representing 27.6%) had arthritis. A little over one in four respondents (25.5%) experienced childhood physical abuse and 5.6% experienced childhood sexual abuse by age 18. In the multivariable regression, respondents who experienced childhood physical abuse had 1.36 times the risk of having arthritis when compared to respondents who did not experience childhood physical abuse (*ARR* = 1.36, *p* < .001, 95% CI = 1.28–1.46). Respondents who experienced childhood sexual abuse had 1.60 times the risk of having arthritis when compared to respondents who did not experience childhood sexual abuse (*ARR* = 1.74, *p* < .001, 95% CI = 1.54–1.97). The findings of this study demonstrate that childhood physical and sexual abuse are associated with arthritis later in adulthood. The associations persisted even after adjusting for demographic, socioeconomic status, body mass index (BMI), current smoking status, and self-perceived physical health. The findings of this study add to the burgeoning number of studies demonstrating the adverse impact of childhood physical and sexual abuse on chronic health outcomes among adults.

## Highlights

• Arthritis has been identified as one of the leading causes of disability in the United States.
• A little over one in four adults (27.6%) had arthritis.
• Respondents who experienced childhood physical abuse had 1.36 times higher risk of having arthritis.
• Respondents who experienced childhood sexual abuse had 1.36 times higher risk of having arthritis.

## 1 Introduction

Arthritis is a chronic inflammatory disease that has been identified as one of the leading causes of disability in the United States (U.S.) (Centers for Disease Control and Prevention (CDC), 2009), and are associated with symptoms like pain, stiffness, tenderness, and swelling of the joints or their surrounding areas (Murphy et al., 2018). Doctor-diagnosed arthritis among the adult US population is projected to increase from 52.5 million to 78.4 million between 2010 and 2040, in part due to the rise in the older adult population (Hootman et al., 2016). Arthritis and other rheumatic conditions can lead to chronic pain, deformity, poor physical functioning, mental health problems, and decreased quality of life over time (Barbour et al., 2013, Barbour et al., 2017, Centers for Disease Control and Prevention, 2017, Khanna et al., 2011, Price et al., 2020). If not treated, arthritis can also increase the risk of mortality (England et al., 2018, Huang et al., 2020, Kiadaliri et al., 2017). Thus, understanding factors associated with arthritis is an important first step towards an effective and efficient allocation of resources in managing arthritis. For the purposes of this study, arthritis was defined to mean having some form of arthritis, rheumatic arthritis, gout, lupus, or fibromyalgia.

One factor that has been identified as contributing to chronic disease is psychosocial stress, which may lead to elevated stress hormones and contribute to inflammatory processes (Cohen et al., 2012, Deighton et al., 2018, Miller et al., 2002). In fact, adverse childhood experiences (ACEs) which include household adversity (e.g., parental incarceration or mental illness), abuse (physical, sexual, emotional), neglect, and separation from caregivers (Felitti et al., 1998) have been found to increase the risk of experiencing mental illness and chronic health conditions later on in life (Afifi et al., 2016, Baiden et al., 2016, Cañizares et al., 2008, Deighton et al., 2018, Dube et al., 2009, Felitti et al., 1998). Cohort studies have also found an association between ACEs and mental health outcomes (Chandan et al., 2019a, Chandan et al., 2019b, Chandan et al., 2019c, Chandan et al., 2020b, Lee et al., 2020, Mersky et al., 2013, Mersky et al., 2018), substance use (Baiden et al., 2014, Ganson et al., 2021), cardiovascular diseases (Chandan et al., 2020c, Rafiq et al., 2020), asthma (Panisch et al., 2021), chronic pain (Stickley et al., 2015), and somatic and visceral pain syndromes (Chandan et al., 2019c). Systematic reviews and meta-analytic studies have also established an association between ACEs and mental health (Bellis et al., 2019), obesity (Schroeder et al., 2021), pain and functional limitations (Bussières et al., 2020), cardiovascular disease (Su et al., 2015), as well as autoimmune conditions such as chronic fatigue syndrome (Borsini et al., 2014, Chandan et al., 2019b) and irritable bowel syndrome (Chitkara et al., 2008).

With respect to the association between ACEs and arthritis, Fuller-Thomson et al. (2016) analyzed data from the 2012 Canadian Community Health Survey-Mental Health, and found that adults with arthritis had higher odds of making a suicide attempt than those in the general population, and this risk was amplified among those with arthritis who also had a history of ACEs. Other studies have also found an association between ACEs and arthritis (Dube et al., 2009, Kopec and Sayre, 2004, Von Korff et al., 2009). ACEs are hypothesized to affect chronic health outcomes including arthritis through increased production of proinflammatory cytokines interleukin-6 and C-reactive protein (Coelho et al., 2014, Danese et al., 2007). A meta-analysis conducted by Baumeister et al. (2016) found that ACEs have the potential to disrupt the regulatory function of hypothalamic–pituitaryadrenal (HPA) which is responsible for the regulation of inflammatory activity in the brain.

Other factors found to be associated with arthritis include female gender (Coffey et al., 2019, Spitzer et al., 2013), older age (Hunter et al., 2017, Jafarzadeh and Felson, 2018), low socioeconomic status (SES), and poor physical health (Barbour et al., 2017, Cañizares et al., 2008). Health risk behaviors such as smoking (Loxton et al., 2021, Strine et al., 2012), obesity, and unhealthy eating habits (Power et al., 2015, Rehkopf et al., 2016, Schuler et al., 2021) are also more prevalent among individuals with a history of ACEs. Systematic reviews and meta-analyses have also found an association between ACEs and these health risk behaviors (Hemmingsson et al., 2014, Palmisano et al., 2016). It has also been well established that autoimmune inflammatory conditions, including arthritis, are associated with health risk behaviors such as obesity (Centers for Disease Control and Prevention, 2011, Crowson et al., 2013, de Hair et al., 2013) and smoking (Blagojevic‐Bucknall et al., 2019, Sugiyama et al., 2010, Torrente-Segarra et al., 2018).

While most ACE literature has maintained that ACEs are interrelated and co-occur (Baiden et al., 2015, Dong et al., 2004, Dube et al., 2009), some scholars suggest that different adversities may have a different impact on health and mental health outcomes (Armiento et al., 2016, Baiden et al., 2017, Brinker and Cheruvu, 2017, Campbell et al., 2018). For instance, one study found that ACE items were better reflected as a three-factor solution, with childhood physical abuse (CPA) and childhood sexual abuse (CSA) separated from items of household dysfunction, such as parental substance use or mental health issues (Ford et al., 2014). In particular, Scott et al. (2011) examined data from 10 countries participating in the World Health Organization (WHO) World Mental Health (WMH) Surveys and found that both CPA and CSA were significantly associated with the onset of osteoarthritis, chronic back and neck pain, and frequent or severe headache, even after controlling for age, sex, country, and smoking status. The study further revealed that CPA and CSA demonstrated the strongest association when compared to all the other childhood adversities (Scott et al., 2011). Campbell et al. (2018) in a longitudinal study examined the differential impact of ACEs on the development of pre-diabetes among adults in the US and found that, of the ACEs items, CPA and CSA were significantly associated with elevated blood fasting insulin levels and higher insulin resistance.

Drawing on a large representative dataset from 17 states from the U.S., the objective of this study was to investigate the extent to which CPA and CSA may be associated with arthritis among adults. We hypothesized that controlling for the effects of other risk factors, CPA or CSA will be associated with arthritis. Examining the association between CPA, CSA, and arthritis could support further research into understanding the mechanisms through which childhood abuse contributes to the development of arthritis in adulthood.

## 2 Materials and methods

### 2.1 Data source and sample

Data for this study were derived from the public use data files of the 2019 Behavioral Risk Factor Surveillance System Survey (BRFSS). The BRFSS is a cross-sectional survey designed and conducted by the Centers for Disease Control and Prevention (CDC) to collect data on health-related risk behaviors, chronic health conditions, and use of preventive services related to the leading causes of death and disability from non-institutionalized adult population (≥18 years) residing in the U.S. (CDC, 2019). A standardized questionnaire is administered to a representative sample of adults by landline telephone with majority of the interviews conducted by cell phone. Detailed information on the study design of the 2019 BRFSS, including the objectives, methodology, and sampling procedure, are available from the U.S. Department of Health and Human Services (CDC, 2019). The study protocol for conducting the 2019 BRFSS was approved by the CDC’s Institutional Review Board (IRB) and the de-identified data are publicly available. The current study was exempted from IRB approval by the lead author’s institution as the data had already been de-identified and did not contain any personal information. The BRFSS questionnaire consists of a core component, optional modules, and state-added questions. The 2019 optional survey module on ACEs was answered by all respondents aged 18 and older in 17 states. The initial sample size for the 2019 BRFSS was 418,268. However, the analysis presented in this study is restricted to respondents aged 18 years and above from the 17 States that collected information on childhood physical and sexual abuse and had data on all the variables included in the analysis. This resulted in a final analytic sample size of 76,016 respondents. Missing data analysis conducted revealed that with the exception of income, missing data on all the variable included in the analysis was<4%. We followed Strengthening the Reporting of Observational Studies in Epidemiology (STROBE) guidelines in the conduct of this study (Von Elm et al., 2007).

### 2.2 Variables

#### 2.2.1 Outcome variable

The outcome variable of interest examined in this paper was arthritis, and it was measured as a binary variable. Survey respondents were asked, “Has a doctor, nurse, or other health professionals ever told you that you had some form of arthritis, rheumatoid arthritis, gout, lupus, or fibromyalgia?” Respondents who answered “yes” were coded as 1, whereas respondents who answered “no” were coded as 0. This item has been found to provide reliable overall arthritis surveillance with adequate sensitivity and specificity (Bombard et al., 2005, Sacks et al., 2005) and has been used in other population-based studies (Barbour et al., 2013, Barbour et al., 2017, Fontaine et al., 2007, Guglielmo et al., 2018, Hootman et al., 2016).

#### 2.2.2 Explanatory variables

The main explanatory variables examined in this paper were CPA and CSA and were both measured as binary variables. These were measured based on items from the original CDC ACE module that asked respondents about events that may have happened to them before they turned 18 years old. CPA was measured based on response to the question, “Not including spanking, (before age 18), how often did a parent or adult in your home ever hit, beat, kick, or physically hurt you in any way?” CSA was measured based on response to the question, “How often did anyone at least 5 years older than you or an adult, force you to have sex?” Responses to each question was coded as “1 = Never”, “2 = Once”, and “3 = More than once.” For the purposes of this study, respondents who indicated “once” and “more than once” were coded as 1, whereas respondents who indicated “never” were coded as 0.

#### 2.2.3 Control variables

Control variables examined in this paper included age, sex, race/ethnicity, marital status, education, income, BMI, current smoking status, and self-perceived physical health. Age was measured as a nominal variable into 18–34 years, then by decade to 65–74 years. Sex of respondent was coded as a binary variable with male as the reference category. Race/ethnicity was measured as a nominal variable into the following categories “Non-Hispanic White”, “Non-Hispanic Black”, “Hispanic”, “Multiracial”, and “Other race/ethnicity.” Marital status was measured as a nominal variable into the following categories: “Married”, “Divorced/separated”, “Widowed”, and “Single/never married.” Education was categorized into “High school education or less”, “Attended college or technical school”, and “Graduated from college or technical school.” Annual household income was measured as an ordinal variable into the following categories “Less than $20,000”, “$20,000–$34,999”, “$35,000–49,999”, “$50,000–$74,999”, and “$75,000 and above.” Respondents with missing data on income were coded as a separate category in order to retain them in the analysis. BMI was measured by dividing self-reported weight in kilograms by self-reported height in meters squared. BMI values were categorized into underweight (BMI < 18.5), normal weight (BMI = 18.5–24.99), overweight (BMI = 25–29.99) and obese (BMI ≥ 30) with normal weight as the reference category. Smoking status was coded as a nominal variable into “0 = Never”, “1 = Former smoker”, and “2 = Current smoker.” Self-perceived physical health was originally measured as an ordinal variable on a five-point Likert scale ranging from excellent to poor. However, this variable was dichotomized by collapsing excellent, very good, and good into one category, called “good” (score = 0), whereas fair and poor were collapsed together into a category called “poor” (score = 1).

### 2.3 Data analyses

Data were analyzed using bivariate and multivariable analytic techniques. First, the distribution of the outcome variable (i.e., arthritis) and explanatory and control variables was examined using percentages. Bivariate associations between arthritis and the categorical variables were also examined using Pearson chi-square test of association. The main analysis involves the use of log-binomial regression to examine the association between CPA and CSA and the relative risks (RRs) of having arthritis while simultaneously controlling for the effects of demographic factors, socioeconomic status variables, BMI, current smoking status, and self-rated physical health (Chen et al., 2014, Lumley et al., 2006). Three regression models were fitted. In Model 1, we regressed arthritis on CPA and CSA. In Model 2, we regressed arthritis on CPA and CSA plus demographic and socioeconomic status variables. In Model 3, we regressed arthritis on CPA and CSA while simultaneously controlling for the effects of demographic factors, socioeconomic status variables, BMI, current smoking status, and self-rated physical health. All the variables were entered into the regression models using the enter procedure (i.e., entering all variables at the same time). The RRs are reported together with their 95% Confidence Intervals (C.I.). Variables were considered significant if the *p-*value was <0.05. Stata’s “svyset” command was used to account for the weighting and complexity of the sampling design employed by the BRFSS. All analyses were performed using Stata version 14.

## 3 Results

### 3.1 Sample characteristics

Table 1 shows the general distribution of the study variables as well as the bivariate association between the study variables by arthritis. Of the 75,717 respondents examined, 20,938 (representing 27.6%) had arthritis. A little over one in four respondents (25.5%) experienced CPA and 5.6% experienced CSA by age 18. More than a third of respondents who experienced CPA (33.7%) compared to 25.6% of respondents who did not experience CPA had arthritis (χ^2^(1) = 468.83, *p* < .001). A little over 42% of respondents who experienced CSA compared to 26.8% of respondents who did not experience CSA had arthritis (χ^2^(1) = 480.72, *p* < .001). Age was positively associated with arthritis with more than half (52.4%) of respondents aged 65–74 years compared to 6.7% of respondents aged 18–35 years having arthritis (χ^2^(4) = 10112.37, *p* < .001). The proportion of females that reported having arthritis (30.7%) was significantly greater than the proportion of males that had arthritis (24.7%; (χ^2^(1) = 340.15, *p* < .001).

**Table 1:** Sample characteristics (n = 75,717).

Variables			Frequency (Weighted %)	% with Arthritis	Chi-square (*p*-value)
**Outcome variable**					
	Has arthritis				
		No	54,779 (72.4)		
		Yes	20,938 (27.6)		

**Main explanatory variables**					
	Childhood physical abuse				468.83 (*p* < .001)
		No	56,379 (74.5)	25.6	
		Yes	19,338 (25.5)	33.7	
	Childhood sexual abuse				480.72 (*p* < .001)
		No	71,457 (94.4)	26.8	
		Yes	4,260 (5.6)	42.3	

**Demographic and control variables**					
	Age				10112.37 (*p* < .001)
		18–34 years	21,188 (28.0)	6.7	
		35–44 years	12,772 (16.9)	17.5	
		45–54 years	13,348 (17.6)	29.6	
		55–64 years	15,798 (20.8)	42.5	
		65–74 years	12,611 (16.7)	52.4	
	Sex				340.15 (*p* < .001)
		Male	38,080 (50.3)	24.7	
		Female	37,637 (49.7)	30.7	
	Race/ethnicity				556.34 (*p* < .001)
		Non-Hispanic White	55,221 (72.9)	29.6	
		Non-Hispanic Black	10,400 (13.7)	25.6	
		Hispanic	6,476 (8.6)	18.0	
		Multiracial	891 (1.2)	28.8	
		Other	2,729 (3.6)	18.0	
	Marital status				3996.41 (*p* < .001)
		Married	40,946 (54.0)	29.7	
		Divorced/Separated	11,245 (14.9)	38.8	
		Widowed	3,386 (4.5)	53.1	
		Single/Never married	20,140 (26.6)	13.1	
	Highest level of education				505.09 (*p* < .001)
		High school or less	30,720 (40.6)	31.3	
		Attended college or technical school	24,414 (32.2)	27.6	
		Graduated from college or technical school	20,583 (27.2)	22.2	
	Income				1336.14 (*p* < .001)
		Less than $20,000	9,750 (12.9)	40.0	
		$20,000-$34,999	11,787 (15.6)	31.8	
		$35,000-$49,999	8,802 (11.6)	29.1	
		$50,000-$74,999	11,222 (14.8)	26.7	
		$75,000 and above	24,621 (32.5)	21.3	
		Missing	9,535 (12.6)	26.2	
	BMI				1943.379 (*p* < .001)
		Normal	21,506 (28.4)	19.1	
		Underweight	1,368 (1.8)	22.6	
		Overweight	25,964 (34.3)	25.7	
		Obese	26,879 (35.5)	36.7	
	Current smoking status				1807.82 (*p* < .001)
		Never	42,939 (56.7)	21.6	
		Former smoker	18,715 (24.7)	36.4	
		Current smoker	14,063 (18.6)	34.4	
	Self-perceived physical health				5136.51 (*p* < .001)
		Good	61,717 (81.5)	22.1	
		Poor	14,000 (18.5)	52.0	

### 3.2 Multivariable regression showing the risk of having arthritis

In the multivariable log-binomial regression, we controlled for the effect of other explanatory variables on arthritis. Table 2 shows the results of the multivariable log-binomial regression results examining the association between CPA and CSA and arthritis. In Model 1, respondents who experienced CPA had 1.38 times the risk of having arthritis (*ARR* = 1.38, *p* < .001, 95% CI = 1.31–1.46) and respondents who experienced CSA had 1.76 times the risk of having arthritis (*ARR* = 1.76, *p* < .001, 95% CI = 1.58–1.97). These significant associations persisted with the addition of demographic factors and socioeconomic status variables in Model 2, and BMI, and self-rated physical health in Model 3. Controlling for the effects of other factors, respondents who experienced CPA had 1.36 times the risk of having arthritis when compared to respondents who did not experience CPA (*ARR* = 1.36, *p* < .001, 95% CI = 1.28–1.46). Respondents who experienced CSA had 1.74 times the risk of having arthritis when compared to respondents who did not experience CSA (*ARR* = 1.74, *p* < .001, 95% CI = 1.54–1.97).

**Table 2:** Multivariable log-binomial regression results predicting arthritis (n = 75,717).

Variables		Model 1		Model 2		Model 3	
		ARR (95% C.I.)	*p*-value	ARR (95% C.I.)	*p*-value	ARR (95% C.I.)	*p*-value
Childhood physical abuse (No)							
	Yes	1.38 (1.30–1.46)	<0.001	1.44 (1.35–1.54)	<0.001	1.36 (1.28–1.46)	<0.001
Childhood sexual abuse (No)							
	Yes	1.76 (1.58–1.97)	<0.001	1.89 (1.68–2.14)	<0.001	1.74 (1.54–1.97)	<0.001
Demographic and control variables							
Age (18–34 years)							
	35–44 years			2.81 (2.47–3.21)	<0.001	2.49 (2.18–2.84)	<0.001
	45–54 years			5.43 (4.80–6.14)	<0.001	4.85 (4.29–5.49)	<0.001
	55–64 years			9.20 (8.16–10.38)	<0.001	8.46 (7.50–9.56)	<0.001
	65–74 years			13.50 (11.97–15.23)	<0.001	12.79 (11.33–14.44)	<0.001
Sex (Male)							
	Female			1.26 (1.18–1.33)	<0.001	1.33 (1.25–1.41)	<0.001
Race/ethnicity (Non-Hispanic White)							
	Non-Hispanic Black			0.84 (0.77–0.92)	<0.001	0.82 (0.75–0.90)	<0.001
	Hispanic			0.63 (0.51–0.76)	<0.001	0.64 (0.53–0.79)	<0.001
	Multiracial			1.12 (0.91–1.39)	0.284	1.11 (0.90–1.36)	0.337
	Other			0.68 (0.56–0.82)	<0.001	0.74 (0.61–0.89)	0.002
Marital status (Married)							
	Divorced/Separated			1.05 (0.97–1.13)	0.272	1.02 (0.94–1.11)	0.575
	Widowed			1.08 (0.97–1.20)	0.161	1.04 (0.93–1.16)	0.480
	Single/Never married			0.74 (0.67–0.82)	<0.001	0.75 (0.68–0.83)	<0.001
Education (High school or less)							
	Attended college or technical school			0.95 (0.88–1.02)	0.172	0.97 (0.90–1.05)	0.499
	Graduated from college or technical school			0.72 (0.67–0.77)	<0.001	0.81 (0.75–0.88)	<0.00
Income (Less than $20,000)							
	$20,000-$34,999			0.67 (0.60–0.74)	<0.001	0.68 (0.61–0.75)	<0.001
	$35,000-$49,999			0.60 (0.54–0.68)	<0.001	0.61 (0.55–0.69)	<0.001
	$50,000-$74,999			0.53 (0.48–0.60)	<0.001	0.55 (0.49–0.62)	<0.001
	$75,000 and above			0.43 (0.38–0.48)	<0.001	0.46 (0.41–0.53)	<0.001
	Missing			0.53 (0.48–0.59)	<0.001	0.57 (0.51–0.64)	<0.001
BMI (Normal)							
	Underweight					0.87 (0.68–1.10)	0.243
	Overweight					1.31 (1.20–1.41)	<0.001
	Obese					2.03 (1.89–2.19)	<0.001
Current smoking status (Never)							
	Former smoker					1.28 1.19–1.37)	<0.001
	Current smoker					1.46 (1.35–1.59)	<0.001
Self-perceived physical health (Good)							
	Poor					2.54 (2.36–2.73)	<0.001

*Note:* Reference category is indicated in parenthesis. ARR indicates adjusted relative risk.

Compared to respondents who self-identified as non-Hispanic Whites, those who self-identified as non-Hispanic Black had an 18% reduction in the risk of having arthritis (*ARR* = 0.82, *p* < .001, 95% CI = 0.75–0.90); those who self-identified as Hispanic had a 36% reduction in the risk of having arthritis (*ARR* = 0.64, *p* < .001, 95% CI = 0.53–0.79); and those self-identifying as other race/ethnicity had a 26% reduction in the risk of having arthritis (*ARR* = 0.74, *p* = .002, 95% CI = 0.61–0.89). Respondents who are single/never married had a 25% reduction in the risk of having arthritis (*ARR* = 0.75, *p* < .001, 95% CI = 0.68–0.83) when compared to their married counterparts. Respondents who were college or technical school graduates, or had a higher income had a reduction in the risk of having arthritis. The risk of having arthritis was 1.31 times as high for respondents who were overweight (*ARR* = 1.31, *p* < .001, 95% CI = 1.20–1.41) and 2.03 times as high for respondents who were obese (*ARR* = 2.03, *p* < .001, 95% CI = 1.89–2.19), both when compared to their counterparts with normal BMI. Compared to respondents who never smoke, the risk of having arthritis was 1.28 times as high for respondents who are former smokers (*ARR* = 1.28, *p* < .001, 95% CI = 1.19–1.37) and 1.46 times as high for respondents who currently smoke (*ARR* = 1.46, *p* < .001, 95% CI = 1.35–1.59). Respondents who perceived their physical health to be poor had 2.54 times the risk of having arthritis when compared to those who perceived their physical health to be good (*ARR* = 2.54, *p* < .001, 95% CI = 2.36–2.73).

## 4 Discussion

The primary objective of this study was to investigate the association between CPA and CSA and self-reported arthritis among adults from 17 states from the U.S. We found that a little over one in four adults (27.6%) had arthritis, and 25.5% experienced CPA, while 5.6% experienced CSA by age 18. The finding that 27.6% of adults had arthritis is consistent with the high prevalence of arthritis reported in prior national reports (Barbour et al., 2013, Barbour et al., 2017) although slightly higher than some other studies that relied on earlier data (Fontaine et al., 2007, Hootman et al., 2016, Jafarzadeh and Felson, 2018). The high proportion of respondents answering yes to having arthritis could be due to episodes of gout. Some recent studies using data from the National Health and Nutrition Examination Survey have observed an increase in the prevalence of gout in the US (Chen‐Xu et al., 2019, Singh et al., 2019). Also, it should be noted that although the 2019 BRFSS is nationally representative, the high prevalence of arthritis found in this sample could be a reflection of the fact that this study focuses only on states that administered the ACEs module. Researchers have found the incidence of arthritis to be much higher among individuals with a history of ACEs than the general population (Kopec and Sayre, 2004).

There is considerable evidence supporting the link between ACEs and health and mental health outcomes later in adulthood (Afifi et al., 2016, Baiden et al., 2015, Cañizares et al., 2008, Deighton et al., 2018, Dong et al., 2004, Dube et al., 2009, Felitti et al., 1998). Yet, only a few studies have specifically examined the association between CPA and CSA, and arthritis among adults (Dube et al., 2009, Kopec and Sayre, 2004, Von Korff et al., 2009), although prior research has examined specific relationships between ACEs and rheumatoid arthritis measured more broadly. Studies conducted with children have revealed that those exposed to ACEs had substantially elevated odds of having rheumatoid arthritis, as well as worse associated physical difficulties, as opposed to either having another type of chronic physical health condition or no health conditions, when compared to their peers with no history of ACEs exposure (Rubinstein et al., 2020). Among adults, however, conflicting findings exist. In one study, although ACEs were associated with susceptibility to rheumatoid arthritis, prevalence rates were not significantly different between adults exposed to ACEs and those in the general population (Luiz et al., 2018). However, over 75% of adults with rheumatoid arthritis affirmed a history of ACEs in a 2014 study by Lisitsyna et al. (2014). Other researchers have also examined the association between ACEs and related conditions such as fibromyalgia (Chandan et al., 2020a, Chandan et al., 2019c, Fuller-Thomson et al., 2011, Jiao et al., 2015, Miró et al., 2020, Ortiz et al., 2016, Tietjen et al., 2010).

In the fully adjusted model, we observed that respondents who experienced CPA had 1.35 times the risk of having arthritis and respondents who experienced CSA had 1.68 times the risk of having arthritis when compared to their counterparts with no such abuse. Our results replicate findings in prior research that suggests an association between adverse childhood events and arthritis among adults (Dube et al., 2009, Kopec and Sayre, 2004, Von Korff et al., 2009). These results are further reflected by findings in one recent review indicating that there is growing interest and awareness of the potential impact of early childhood and chronic stress in arthritis development (Schwetlik et al., 2020). Whereas previous research has focused on establishing a dose–response relationship between ACEs related to maltreatment and household dysfunction and adult-onset of arthritis (Von Korff et al., 2009), our findings extend the literature in this field by focusing specifically on CPA and CSA that may have more of an impact on arthritis than other ACE categories such as parental substance use or mental health issues. One possible reason that may contribute to the association between CPA and CSA and arthritis could be prolonged stress and its impact on the HPA leading to dysregulation of the immune system. Indeed, it has been postulated that during chronic stress, the HPA remains hyper-activated, resulting in chronically elevated cortisol levels which may lead to long-term systemic damage (Baumeister et al., 2016, Cohen et al., 2012, Danese et al., 2007).

After adjusting for demographic variables and SES, we found that the incidence of arthritis was higher among older-aged adults, females, non-Hispanic Whites, individuals with higher BMI, or those with low self-rated health. In contrast, the incidence of having arthritis was lower among individuals with higher education or individuals who had higher income. These findings corroborate previous research that has found associations between demographic factors such as age (Hunter et al., 2017, Jafarzadeh and Felson, 2018), gender (Coffey et al., 2019, Spitzer et al., 2013), low SES, and poor physical health (Barbour et al., 2017, Cañizares et al., 2008) with the development of inflammatory disorders such as arthritis. Thus, our results are consistent with the literature regarding factors that may predispose adults to the development of arthritis.

Additionally, we observed that modifiable lifestyle factors such as smoking and obesity were associated with arthritis. Past studies have also reported similar findings (Crowson et al., 2013, de Hair et al., 2013, Sugiyama et al., 2010). One proposed mechanism to explain the link between smoking and obesity with arthritis is the increasingly emerging evidence that both obesity and smoking may stimulate low-grade inflammation and immune dysregulation resulting in elevated pro-inflammatory cytokines and subsequent joint damage (Ouchi et al., 2011).

The findings of the current study emphasize the potential long-term influence of CPA and CSA on physical health outcomes, namely arthritis, in adulthood. Given the socioeconomic and health burden associated with a chronic illness such as arthritis, it is important for additional research to be conducted that explores the complex interplay between these psychosocial risk factors such as CPA and CSA and the biological mechanisms that are associated with arthritis and related chronic health conditions. Our study highlights the importance of addressing proximal factors such as experiences of CPA and CSA that may contribute to the development of arthritis. Incorporating CPA and CSA as risk factors into strategies designed to prevent arthritis at the population level is important. In addition, previous literature suggests potential for a cyclical and intergenerational transmission of CPA and CSA (Larkin et al., 2012), thus intervention efforts to address childhood abuse and its associated outcomes should be robust and include parenting programs.

### 4.1 Study limitations and strengths

A major strength of this study includes the use of a large dataset, which ensures that our study is sufficiently powered to detect differences. Also, the use of weights enables the generalization of the study results to the US adult population in 17 states that administer the optional ACEs module in the 2019 BRFSS. However, the following limitations are worth noting. First, this was a cross-sectional study and thus precluded our ability to draw any causal inferences between the study variables. For instance, it was not possible to ascertain the exact timing and duration of CPA and CSA, and arthritis from this study. It is possible that some adults may have been diagnosed of having arthritis in childhood prior to them experiencing CPA or CSA. Also, we do not preclude the possibility of reverse causality between arthritis and the covariates, in particular self-perceived physical health and smoking status. However, given past literature supporting that poor health status and health-compromising behaviors are associated with arthritis, adjusting for health status and health-compromising behaviors is important to prevent the underestimation of the model and ascertain the true association between CPA/CSA and arthritis. Second, given that the survey utilizes self-reported information to ascertain arthritis and adverse childhood experiences (CPA and CSA), there is the possibility of recall bias. Another limitation of the study is the lack of specification of type of arthritis and related conditions. Given that arthritis was measured using a single item in the survey, it was not possible to investigate associations between CPA and CSA and specific types of arthritis and related conditions such as rheumatoid arthritis, gout, lupus, or fibromyalgia. However, prior research indicates that self-reports of arthritis correlate well with clinical diagnosis (Sacks et al., 2005). Fourth, the use of secondary data limits our ability to investigate other theoretically known confounders of CPA, CSA, and arthritis, such as family history of arthritis and environmental exposures. Finally, we limited our analytic sample to respondents in the 17 States that collected information on CPA and CSA.

### 4.2 Conclusion

In conclusion, the findings of the current study demonstrate that CPA and CSA are associated with arthritis later in adulthood. The associations persisted even after adjusting for demographic, socioeconomic status, BMI, and self-perceived physical health. Our findings add to the burgeoning number of studies demonstrating the adverse impact of CPA and CSA on chronic health outcomes among adults. Our results underscore the importance of using a multidisciplinary approach that integrates biological evidence with knowledge of psychosocial risk factors such as early life adversity into a deeper understanding of complex mechanisms that are associated with arthritis. Future researchers are encouraged to pursue this approach and incorporate their findings into prevention strategies meant to attenuate the risk of arthritis among adults with a history of ACEs.

## CRediT authorship contribution statement

**Philip Baiden:** Conceptualization, Methodology, Investigation, Data curation, Formal analysis, Writing - review & editing. **Lisa S. Panisch:** Writing - original draft, Writing - review & editing. **Henry K. Onyeaka:** Writing - original draft, Writing - review & editing. **Catherine A. LaBrenz:** Writing - review & editing. **Yeonwoo Kim:** Writing - review & editing.

## Declaration of Competing Interest

The authors declare that they have no known competing financial interests or personal relationships that could have appeared to influence the work reported in this paper.
